# Reply to Walker and Nott

**DOI:** 10.1016/j.bjao.2025.100423

**Published:** 2025-07-24

**Authors:** Anton Utas, Stefanie Seifert, Knut Taxbro

**Affiliations:** 1 Department of Anaesthesia and Intensive Care Medicine, Ryhov County Hospital, Jönköping, Sweden; 2 Department of Biomedical and Clinical Sciences, Linköping University, Linköping, Sweden

Editor

We would like to thank Professors Walker and Nott for their insightful and constructive correspondence regarding our *post hoc* analysis of the PICCPORT trial.^1^ Their letter highlights several important considerations that underscore the complexity of vascular access decisions in oncology care—particularly when viewed through the lens of diverse healthcare systems and clinical practices.

We fully agree that vascular access is a critical component of cancer care, and that practices inevitably vary across countries, healthcare systems, and even between public and private sectors within the same country. The Australian perspective they provide is a valuable contribution to this ongoing discussion.

An initiative currently led by Associate Professor Peter J. Carr and PhD scholar Caitríona Duggan at the University of Galway in Ireland has conducted a pan-European survey aimed at characterising vascular access device (VAD) practices for systemic anticancer therapy across Europe. This effort addresses key gaps in clinician decision-making, training, and complication management. The results of this initiative are expected to be published in the near future.

To date, no randomised controlled trials addressing vascular access in cancer care have been conducted on an international scale. While the CAVA trial remains the largest of its type, it was limited to the UK,^2^ highlighting the need for broader, multinational efforts to capture the diversity of clinical practice and patient experience globally.

As Walker and Nott rightly point out, current practice is shaped by a range of factors—including healthcare financing models, availability of trained personnel, and the limitations of existing guidelines. The absence of clear, device-specific recommendations in current guidelines has contributed to variability in practice. Initiatives such as The Michigan Appropriateness Guide for Intravenous Catheters (MAGIC) project, led by Professor Vineet Chopra’s group in the USA, are commendable efforts to address this gap and provide more structured guidance.^3^ The MAGIC-ONC project recently convened an international, multidisciplinary panel and used the RAND/UCLA Appropriateness Method to develop recommendations for VAD selection, insertion, and management in patients with cancer. A systematic literature review informed the development of clinical scenarios, which were rated by the panel for appropriateness based on cancer type, treatment indication, urgency, and anticipated duration of use. These recommendations are currently undergoing pre-publication preparations.

We also appreciate the emphasis placed on patient experience, particularly regarding pain during insertion. This is a crucial issue, especially for vulnerable patients undergoing cancer treatment. There is certainly room for further innovation and standardisation in this area to improve patient satisfaction and reduce procedural discomfort. Our group is currently addressing these challenges, and results from a full-scale randomised trial evaluating the effectiveness of patient-controlled sedation during PORT implantation (PACSPI trial) are expected to be published soon.

Both the CAVA and PICCPORT trials addressed the health economic dimensions of VAD selection in cancer care. Although both were conducted in European contexts, their findings are aligned.^4^ ^5^ Taken together—considering complications, cost, and patient-reported outcomes—PORTs appear to offer advantages over other eligible VADs for systemic anticancer therapy.^6^

## Declarations of interest

The authors declare that they have no conflicts of interest.
